# WTC Five-Year Assessment: Herbert et al. Respond

**Published:** 2007-02

**Authors:** Robin Herbert, Gwen Skloot, Kristina Metzger, Philip J. Landrigan, Jacqueline Moline, Diane Stein, Andrew Todd, Stephen M. Levin, Sherry Baron, Iris Udasin

**Affiliations:** Mount Sinai School of Medicine, New York, New York, E-mail: robin.herbert@mssm.edu; National Institute for Occupational Safety and Health, Centers for Disease Control and Prevention, Cincinnati, Ohio; Environmental and Occupational Health Sciences Institute, University of Medicine and Dentistry of New Jersey, Piscataway, New Jersey

In our article (Herbert et al. 2006), we described the establishment of the World Trade Center (WTC) Worker and Volunteer Medical Screening Program and presented results of screening examinations undertaken between 2002 and 2004 among a heterogeneous group of 9,442 WTC responders.

Miller expresses concern about the validity of self-reported upper and lower respiratory symptoms in WTC responders. He notes correctly that self-reported symptoms are inherently subjective. However, symptoms cannot merely be dismissed as unimportant, especially when they are persistent and when, as was the case here, the pattern of their occurrence closely parallels severity of exposure. We reported that symptoms were most common among those responders who arrived earliest at the WTC site and who consequently suffered the heaviest exposures to the highest levels of dust and smoke (Herbert et al. 2006). This finding has high inherent biological plausibility. To be sure, the potential for recall bias is always present in a symptom-based survey. In reality, however, recall bias could be of concern only if we had reason to believe that responders in different exposure groups recalled past and current symptomatology differently. Finally, to further ensure the validity of our findings, we buttressed our assessment of symptoms with chest X rays and pulmonary function tests.

Miller also expresses concern that objective results were “confined to spirometry, which does not provide insight into all aspects of respiratory impairment.” Although we recognize the limitations of spirometry, a large-scale screening program has practical restrictions in testing that can be accomplished. In fact, in Miller’s own 1991 survey of a population 10 times smaller than our own (Miller et al. 1991), only spirometry was used as a screening tool. Miller observes that our results were “unlike virtually all spirometric surveys of a large population” since there was “little difference in impairment by smoking status.” We would agree with Miller that our population was distinct by the very nature of the exposures involved and that this should be considered in evaluating the lack of difference in impairment based on smoking status. One speculation is that the overwhelming exposure to toxic chemicals at the WTC disaster may have masked differences between smokers and nonsmokers.

Miller erroneously states that most spirometric impairments were classified as “restrictive.” We were quite careful not to use this term because it cannot be confirmed by spirometry alone. Instead we chose the designation of low forced vital capacity (FVC) (Herbert et al. 2006). Like Miller, we were surprised by this finding as well as by the observation that fewer responders had reversible airway obstruction, which would have confirmed asthma in those with asthma-like symptoms. However, asthma is by its very nature intermittent, and spirometry tests are only a “snapshot in time,” so normal spirometry results do not rule out asthma. Unfortunately, we were unable to provide inhalation challenge tests for the cohort because of the constraints of conducting a large multicenter clinical screening program.

We listed the many possible reasons for a high prevalence of a low FVC in the “Discussion” of our article (Herbert et al. 2006). One member of our working group (G.S.) is currently leading an initiative to estimate the individual contribution of each of these factors by describing the results of additional diagnostic procedures not included in routine screening.

Examinations of the WTC population continue and are expected to proceed for many years to come. As of 31 December 2006, we have examined > 18,500 WTC responders and provided follow-up examinations to > 7,000. We expect to report on findings from those examinations within the next year. In addition, we will be reporting further on the relationship between symptoms and screening spirometry. These analyses should provide further insight into the potential pulmonary impairment of individuals exposed at the WTC disaster.
